# Anemia levels in the preconception period and the first trimester of pregnancy: a national, multicentric and cross-sectional study

**DOI:** 10.61622/rbgo/2025rbgo10001

**Published:** 2025-02-13

**Authors:** Aytaj Jafarzade, Veli Mi̇hmanli, And Yavuz, Murat Akbaş, Gürcan Türkyilmaz, Esra Nur Özkan, Murat İbrahim Toplu, Yücel Kaya, Damla Yasemin Yenli̇k Kaya, Mustafa Yildiz, Ali Emre Ati̇k, Elif İlgazi̇ Kiliç, Burcu Özata, Sehtap Nazlı Kiliç Çeti̇n, Berk Bulut, Halide Gül Okuducu Aydin, Lale Aslanova, Çağdaş Nurettin Emekli̇oğlu, Melike Eren, Elif Uçar, Kaan Eray Uzun, Osman Ufuk Eki̇z, Muhittin Tamer Mungan

**Affiliations:** 1 Obstetrics and Gynecology Department Liv Ankara Hospital Ankara Turkey Obstetrics and Gynecology Department, Liv Ankara Hospital, Ankara, Turkey.; 2 Obstetrics and Gynecology Department Cemil Taşçıoğlu City Hospital Istanbul Turkey Obstetrics and Gynecology Department, Cemil Taşçıoğlu City Hospital, Istanbul, Turkey.; 3 Perinatology Department Antalya Training and Research Hospital Antalya Turkey Perinatology Department, Antalya Training and Research Hospital, Antalya, Turkey.; 4 Perinatology Department Izmir Can Hospital Izmir Turkey Perinatology Department, Izmir Can Hospital, Izmir, Turkey.; 5 Perinatology Department Lokman Hekim Hospital Van Turkey Perinatology Department, Lokman Hekim Hospital, Van, Turkey.; 6 Eskişehir Hospital Obstetrics and Gynecology Department Izmir Turkey Eskişehir Hospital, Obstetrics and Gynecology Department, Izmir, Turkey.; 7 Obstetrics and Gynecology Department Esenler Medipol University Hospital Istanbul Turkey Obstetrics and Gynecology Department, Esenler Medipol University Hospital, Istanbul, Turkey.; 8 Obstetrics and Gynecology Department Privat Derman Hospital Kırklareli Turkey Obstetrics and Gynecology Department, Privat Derman Hospital, Kırklareli, Turkey.; 9 Obstetrics and Gynecology Department Gungoren Hospital Istanbul Turkey Obstetrics and Gynecology Department, Gungoren Hospital, Istanbul, Turkey.; 10 Obstetrics and Gynecology Department Kayseri City Hospital Kayseri Turkey Obstetrics and Gynecology Department, Kayseri City Hospital, Kayseri, Turkey.; 11 Obstetrics and Gynecology Department Ordu University Training and Research Hospital Ordu Turkey Obstetrics and Gynecology Department, Ordu University Training and Research Hospital, Ordu, Turkey.; 12 Obstetrics and Gynecology Department Beykent University Hospital Istanbul Turkey Obstetrics and Gynecology Department, Beykent University Hospital, Istanbul, Turkey.; 13 Obstetrics and Gynecology Department Kolan Hospital Istanbul Turkey Obstetrics and Gynecology Department, Kolan Hospital, Istanbul, Turkey.; 14 Obstetrics and Gynecology Department Kocaeli Darica Farabi Training and Research Hospital Kocaeli Turkey Obstetrics and Gynecology Department, Kocaeli Darica Farabi Training and Research Hospital, Kocaeli, Turkey.; 15 Obstetrics and Gynecology Department Afiyet Hospital Istanbul Turkey Obstetrics and Gynecology Department, Afiyet Hospital, Istanbul, Turkey.; 16 Obstetrics and Gynecology Department Karabuk University Training and Research Hospital Karabuk Turkey Obstetrics and Gynecology Department, Karabuk University Training and Research Hospital, Karabuk, Turkey.; 17 Obstetrics and Gynecology Department Ahlat Goverment Hospital Bitlis Turkey Obstetrics and Gynecology Department, Ahlat Goverment Hospital, Bitlis, Turkey.; 18 Obstetrics and Gynecology Department Esenler Privat Hospital Istanbul Turkey Obstetrics and Gynecology Department, Esenler Privat Hospital, Istanbul, Turkey.; 19 Obstetrics and Gynecology Department Bartın Goverment Hospital Bartın Turkey Obstetrics and Gynecology Department, Bartın Goverment Hospital, Bartın, Turkey.; 20 Science Faculty Gazi University Ankara Turkey Statistic Department, Science Faculty, Gazi University, Ankara, Turkey.; 21 Perinatology Department Koru Ankara Hospital Ankara Turkey Perinatology Department, Koru Ankara Hospital, Ankara, Turkey.

**Keywords:** Anemia, iron-deficiency, Pregnancy trimester, first, Preconception care, Pregnancy complications, hematologic

## Abstract

**Objective:**

The study aimed to determine the level of anemia in pregnant women in the first trimester and in the preconception period by conducting nationwide research.

**Methods:**

The study was designed as retrospective, cross-sectional, and multicenter research. A total of 17 centers from 13 provinces were included in the study. The study was conducted with the participation of two groups of patients who applied to the obstetrics polyclinic between 1 January 2023 and 1 July 2023, who were in the first trimester of pregnancy and who were in the preconception period planning pregnancy.

**Results:**

In total 4,265 women were included in the study. Of these women, 3,884 (91%) were in the first trimester of their pregnancy and 381 (9%) were in the preconception period. Anemia was detected in 24.1% (n=1030) of the patients. Of these patients, 20.6% (n=877) were pregnant women in the first trimester and 3.6% (n=153) were in the preconception period. A statistically significant and positive relationship was found between anemia and meat consumption frequency, educational status, and socioeconomic status of the patients (p=0.000, p=0.000, p=0.000). In addition, a statistically significant and negative correlation was determined between anemia and the number of pregnancies and the parity number (p=0.001, p=0.000) in both groups.

**Conclusion:**

Anemia is a public health problem. Anemia has been determined to be an important problem both in the preconception period and early periods of pregnancy. It is necessary to revise the programs and interventions to reduce the prevalence of anemia and redesign them in line with current conditions.

## Introduction

Anemia is a serious global public health problem affecting children, adolescents, and women during pregnancy and after delivery. The World Health Organization (WHO) estimates that 37% of pregnant women and 30% of women in the age range of 15-49 years are anemic.^(1)^A hemoglobin concentration below 12 g/dl for women in reproductive age but not pregnant and breastfeeding women and a hemoglobin concentration below 11 g/dL for pregnant women are defined as anemia by WHO.^(2)^ While anemia in pregnancy is a public health problem, it is known to increase maternal and fetal risks (higher maternal mortality, perinatal death, preeclampsia, and abnormal birth small for gestational age (SGA)).^(3,4)^Anemia especially in the first trimester of pregnancy is associated with preterm birth and low birth weight.^(5)^Although nutritional factors such as Vitamin B12 and folate and nutritional external factors such as inflammation are significant factors in the etiology of anemia, iron deficiency is the leading cause of anemia worldwide, and it is estimated that 50% of all anemia cases result from iron deficiency.^(6,7,8)^An adequate amount of iron is indispensable for brain growth and development in the fetal, neonatal, and childhood periods.

WHO concluded that serum ferritin concentration is a good indicator of iron stores and that it should be used to diagnose iron deficiency in healthy individuals.^(9)^In situations where there is no inflammation, plasma/serum ferritin concentration displays a positive correlation with the size of total body iron stores. Ferritin may increase depending on excessive iron loading or liver diseases and other reasons such as obesity, inflammation, and malignancy.^(10)^ WHO defines iron deficiency as a lower level of ferritin than 15 μg/L in adults in situations not accompanied by inflammation, and in situations accompanied by inflammation, a ferritin level lower than 70 μg/L can indicate iron deficiency.^(9)^However, checking serum ferritin levels to determine iron deficiency anemia in the last trimester of pregnancy may not be useful because there is bone marrow iron in the body in that period.^(11)^ Between weeks 20 and 28 of pregnancy, hemoglobin levels are measured as 2 g/dL lower depending on serum plasma hemodilution.^(12)^There are various physiological changes that occur in pregnancy (increase in acute phase proteins, increase in the second-trimester plasma volume, changes in the inflammatory measurements in the last trimester) that may contribute to changes in the threshold values of iron deficiency expressed by serum ferritin.^(9)^Therefore, it is appropriate to examine ferritin levels in pregnant women in the first trimester to diagnose iron deficiency anemia.^(9)^The number of national studies on the prevalence of anemia in adults in Türkiye is quite limited, but several studies have been conducted in certain regions.^(13,14)^Therefore, in this multi-center and cross-sectional study, anemia levels of the first-trimester pregnant women and women in the preconception period were examined.

The primary aim of the study was to determine the diagnostic accuracy of ferritin concentrations (serum or plasma) in the determination of iron deficiency, and the secondary aim was to determine the relationship between anemia and age, BMI, smoking status, socio-economic status, educational status, and nutrition.

## Methods

In the retrospective, multi-center, and cross-sectional study, 4.265 women who presented to the obstetrics outpatient clinic between 1 January and 1 July 2023 were included in the sample. Female patients who presented to the obstetric outpatient clinic in the period specified above, who were in the first trimester of their pregnancy, and who were in the preconception period were included in the study. In total, 17 centers from 13 provinces of Türkiye participated in the study.

The demographic data of the patients were obtained from the data systems of the hospitals by the researchers. The data of the patients who had complete demographic information were included in the study. The study inclusion criteria were being in the age range of 18-45 years, being mentally healthy, being in the first trimester of pregnancy, and being in the preconception period.

Patients who had chronic gastrointestinal diseases (Chron’s, ulcerative colitis, etc.), who had undergone intestinal resection due to surgical reasons, who had stomach reduction surgery, hematological patients (sickle-cell anemia, leukemia, aplastic anemia, thalassemia, hemolytic anemia, etc.), cancer patients, patients, who had acute infections and who had inflammation-related white blood cell increase (WBC > 10-10^6^), who received anemia treatment in the last 6 months, who received iron in multivitamins in the last 6 months, and rheumatological patients were excluded from the study. The study data obtained from the data systems of the hospitals were recorded on the data collection form developed by the researchers.

In line with the recommendation of the World Health Organization, iron deficiency <12 g/dL in the preconception period, <11 g/dL in the first trimester of pregnancy, and serum ferritin <15.0 μg/L were accepted as anemia and included in the study. In addition, anemia levels in pregnancy were examined in 3 groups according to the recommendation of the World Health Organization: Mild (9–10.9 g/dL), Moderate (7–8.9 g/dL), Severe (<7 g/dL). Inflammation anemia was defined as anemia in the presence of normal serum ferritin concentrations and high inflammatory biomarkers (i.e., Hb < 11.0 g/dL, SF > 15.0 μg/L, plus CRP > 5.0 mg/L).^(15)^ Anemia in non-pregnant women was operationalized as a categorical variable by predefined cut-off points as mild (hemoglobin level 10–11.9 g/dL), moderate (hemoglobin level 7–9.9 g/dL), and severe (hemoglobin level < 7 g/dL) anemia.^(15)^

Ethical approval for the study was obtained from the Gazi University Ethics Committee with the approval number E-77082166-604.01.02-808629.

## Results

A total of 4,265 women were included in the study. Of these women, 3,884 (91%) were pregnant women in the first trimester and 381 (9%) were in the preconception period. 24.1% (n=1030) of the cases were determined to be anemic. Of these patients, 20.6% (n=877) were pregnant women in the first trimester and 3.6% (n=153) were in the preconception period. The demographic characteristics of the patients are presented in table 1.

**Table 1:** Demographic characteristics

Characteristics	Pregnant	Non-Pregnant
Age y mean± SD	29±1.2	30±1.1
Gravide median range	3(0-4)	2(0-3)
Parite median range	2(0-3)	1(0-2)
BMI (kg/m2 ) mean±SD	23±1.4	24±2.1
Smoking, n (%)		
Yes	775	108
No	3,109	273
s-ferritin, mg/L mean±SD	26±2.3	37±1,2
Hemoglobin Mean ±SD	10.0±0.1	10.9±11
Education		
Illiterate	283	7
Primary School	640	34
Middle	770	72
High School	887	127
Univeriste	1,105	131
Socio-economic status		
Low	249	14
Middle	2,597	207
High	1,100	134
Employee	2,116	222
Not working	1,761	158
Frequency of meat consumption (weeks)		
Doesn’t eat meat	444	53
At least once a week	1,183	122
At least >2 per week	2,254	205

**Figure 1:**
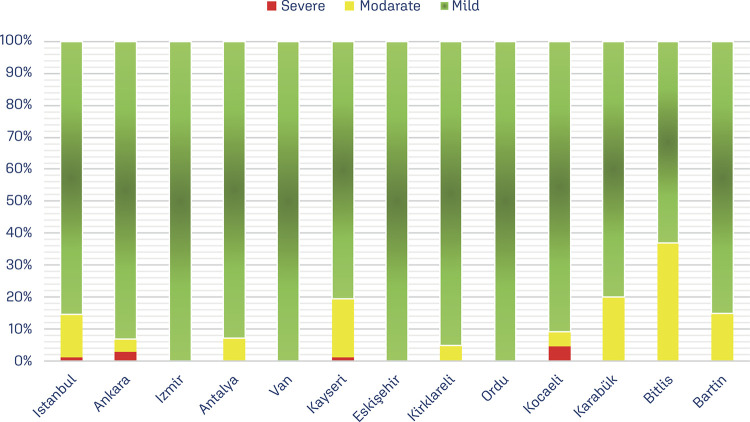
Classification of anemia in pregnant women according to provinces

When the subtypes of anemia were examined in terms of severity, the rate of pregnant women with mild anemia (9–10.9 g/dL) was 18% (n=769), the rate of pregnant women with moderate anemia (7–8.9 g/dL) was 2.6%, (n=114), and the rate of pregnant women with severe anemia (<7 g/dL) was found to be 0.2% (n= 10) (Figure 1).

In the non-pregnant group, the rate of anemia <7 g/dL was 0.02% (n=1), the rate of anemia between 7–9.9 g/dL was 0.4% (n=18), and the rate of anemia between 10–11.9 g/dL was 3% (n=134). When the ferritin and hemoglobin levels of the women in both groups were examined, the mean hemoglobin level in the pregnant anemic group was determined as 9.8 g/dL and the mean ferritin level as 8.86 μg/L, while the mean hemoglobin level in the pregnant non-anemic group was found as 12.64 g/dL and mean ferritin levels as 38.4 μg/L. The mean hemoglobin level of anemic women in the preconception period was 11.7 g/dL and the mean ferritin level was 11.14 μg/L, while the mean hemoglobin level in the non-anemic women in the preconception period was 13 g/dL and the mean serum ferritin level was 39 μg/L (Table 2).

**Table 2:** Ferritin and hemoglobin levels of pregnant and preconceptional patients

	Pregnant		Preconsepsiyonel	
	Anemia Mean ± SD	Non-anemic Mean ± SD	Anemia Mean ± SD	Non-anemic Mean ± SD
Ferritin μg/l Mean ± SD	8,86±3.91	38,14±58,30	11.14±5.36	33.06±31.60
Hemoglobin g/Dl Mean ± SD	8.31 ± 3.82	12.64 ± 3.12	11.7 ± 1.31	13.2 ± 0.74

In total, 32% (n=1370) of the pregnant women and 2.9% (n=124) of the non-pregnant women had ferritin levels below <15 μg/L. The mean age of the pregnant women was 29 ± 1.2 and the mean age of the non-pregnant women was found to be 30 ± 1.1. No statistically significant relationship was found between BMI and anemia (p=0.382). Similarly, no statistically significant relationship was determined between age and anemia (p=0.225). There was a statistically significant correlation between smoking status and anemia. The rate of anemia was found to be statistically significantly higher in the smoking pregnant women compared to non-smokers (p=0.000). There was also a statistically significant relationship between meat consumption frequency and anemia (p=0.000). In other words, as the weekly meat consumption frequency increased, the rate of anemia decreased. When the relationship between employment status and anemia was examined, it was seen that the rate of anemia was statistically significantly higher in employed patients compared to unemployed ones (p=0.008). The socioeconomic status of the patients was categorized as low, moderate, and high. A statistically significant relationship was found between socioeconomic status and anemia (p=0.000). That is, anemia was observed less in individuals with high economic status (both in the pregnant women and the women in the preconception period). Regarding educational status, the patients were categorized as illiterate, primary school, secondary school, high school, and university education levels. A statistically significant correlation was determined between educational level and anemia (p=0.000). Accordingly, anemia was observed less in individuals with high education levels. In addition, there was a statistically significant relationship between employment and anemia. The rate of anemia was lower in employed individuals compared to unemployed ones (p=0.000). Moreover, a statistically significant correlation was determined between anemia and the number of pregnancies and the parity number (p=0.001, p=0.000) in both groups. The rate of anemia was found to be higher in individuals who had a high number of pregnancies and parities.

## Discussion

In the present study, the rate of anemia was found to be 24% in the pregnant women and the women in the preconception period. In the single-center study they conducted in 2016, Saydam et al.^(13)^determined the rate of anemia as 27.8%. While the results of both studies are close to each other, in the present study, the rate of anemia was found to be lower. The reason for this could be the change in eating habits, widespread use of food supplements in the post-COVID-19 pandemic process, and the success of health programs against anemia carried out by the Turkish Ministry of Health (Iron Support in Pregnant Women, Türkiye Like Iron, etc.). However, according to the 2008 anemia classification by the World Health Organization in terms of public health, a rate of 20-39% anemia is in the moderate public health problem category.^(15,16)^This shows that we are still not in the desired position.

In the study they conducted in 2022, Yalcin et al.^(14)^determined the rate of anemia in first-trimester pregnant women as 6.2%. In the present study, we found this rate as 20.6%. However, the study by Yalcin et al.^(14)^ was conducted only in Ankara province (population 5.7 million). In two other studies conducted in Ankara province, first-trimester anemia rates were found as 5.7%^(17)^and 8.8%.^(18)^In the present study, the pregnant anemia rate for Ankara province was 8.9%.

The great majority of the patients included in the study were pregnant women (91%, n=3,884), and a small portion of them were patients who were planning pregnancy and presented to the hospital for a pre-pregnancy checkup. The reason why the number of participants in the preconception period was low is that all centers included in the study were either secondary care or tertiary care hospitals. Patients usually present to primary care health institutions for pre-pregnancy control and consultancy.

When we examine the anemia subtypes of pregnant women, we see that the highest rate of the anemic pregnant women (18%) was in the mild anemia group (9-10.9 g/dL).

In several studies conducted in Türkiye, no statistically significant correlation was found between age and anemia.^(17-19)^ In the study conducted by Elmaghraby et al.,^(20)^different from our study results, they found a statistically significant relationship between anemia and age. In the present study, however, no correlation was found between age and anemia.

Although Launbo et al.^(21)^argued that there was a correlation between anemia and women with high BMI (obese), no statistically significant relationship was found between BMI and anemia either in our study or other studies^(22)^consistent with our results. The reason for this could be that the mean BMI score of the participants was 24, but the mean BMI score was >25 in studies that advocate a relationship between BMI and anemia.

In the present study, a statistically significant relationship was found between anemia and income level, educational level, and employment status. There are studies^(23-25)^whose results are similar to our results. This may have resulted from the fact that women with higher educational and welfare levels can have easier access to nutritious foods and food supplements compared to women with lower educational and welfare levels. As all individuals have easy access to health services in Türkiye independently from welfare status, access to health services in Turkish society is not among the factors affecting anemia. In addition, starting from week 16 of pregnancy, iron support is started in primary care health institutions whether the pregnant woman is anemic or not. Therefore, the causes of anemia should be determined in each country considering eating habits, health policies, and socioeconomic status of that society. There are also studies that found conflicting results with the results of our study.^(13,14)^These two studies were conducted in Turkish society. However, we believe that as these studies were single-center studies, they may have reached different conclusions.

In our study, we found a statistically significant correlation between anemia and the number of pregnancies and parities. As the number of pregnancies, births, and miscarriages increased, the bleeding rates of these women may have increased, which could lead to anemia. Nevertheless, in the study they conducted, Tunç et al.^(26)^could not determine a statistically significant relationship between anemia and the number of pregnancies and parities.

When we examined the relationship between anemia and red meat consumption, we found that the rate of anemia was lower in individuals who consumed more red meat weekly. There are studies whose results support^(17)^and conflict with^(27)^ our study results. Iron intake through diet is an important factor. However, it should be remembered that iron is found not only in red meat but also in many vegetables. It is important to be aware of meat types and vegetables that are rich in iron. On the other hand, we believe that eating habits change every decade and that social media, diet program practices, and diet programs on TV also have a potential effect on anemia.

Besides, in the present study, we found statistically significantly higher rates of anemia in smoking patients compared to non-smokers (p=0.001). In studies supporting the results of our study, results consistent with our results were determined regarding the correlation between smoking status and anemia.^(28)^

The prevalence of anemia among pregnant and non-pregnant women has been included as primary result indicators in the basic indicator set for the “Global Nutrition Monitoring Framework” program of the World Health Organization. These indicators aim to reduce the rate of anemia among women of reproductive age by 50% by 2025 and are used to monitor the progress on the path to reaching Global Nutrition Target 2.^(16)^

When ferritin levels are evaluated along with hemoglobin levels, iron deficiency has been the most reliable and most frequently used non-invasive test in the diagnosis of anemia.^(29,30)^In our study, iron deficiency anemia rate (ferritin <15.0 μg/L) was found to be 32% in pregnant anemic individuals and 2.9% in non-pregnant individuals. Finkelstein et al.^(4)^found first-trimester iron deficiency anemia in pregnancy (ferritin <15.0 μg/L) to be 48%.

Anemia is a public health problem with a high prevalence in underdeveloped and developing countries. Health programs in this regard are organized and implemented. However, eating habits, welfare levels, and food supplement intakes of societies as well as countries’ economic status change over time. New programs should be organized and put into practice considering these factors. Studies that will determine the anemia levels of different segments of society at regular intervals are needed to identify the effect of these factors on society are needed. In the study, we aimed to determine anemia levels in the post-COVID-19 pandemic period.

The limitations of the study are that it had a retrospective nature, no distinction was made between villages and towns affiliated with the provinces, and the number of family members, type of delivery, and the number of miscarriages were not considered. In addition, not including eating habits that lead to anemia and the effect of social media in the study may lead to a bias in the interpretation of the results.

## Conclusion

The study sheds light on the current status of the first trimester and preconception period population of Türkiye. However, we think that further multi-center studies with a prospective design that will encompass all provinces and all segments of society should be conducted.
